
JOURNAL INFORMATION
==============================
NLM Title Abbreviation: ScientificWorldJournal
Journal ID: ScientificWorldJournal
NLM Journal ID: 101131163
Journal ID: TSWJ

The Scientific World Journal

ISSN: 2356-6140
EISSN: 1537-744X
Publisher: Wiley

ARTICLE INFORMATION
==============================
PMCID: PMC5720104
PMID: 21218268
DOI: 10.1100/tsw.2011.14
Article ID: 154612
Article version: 1
Subjects: Directions in Science

Mediation of Immunoregulation by Dendritic Cells

Dasgupta Suryasarathi 1 2 *
1 Channing Laboratory, Department of Medicine, Brigham and Women's Hospital, Harvard Medical School, Boston, USA
2 Department of Microbiology and Molecular Genetics, Harvard Medical School, Boston, USA
*Suryasarathi Dasgupta: sdasgupta@rics.bwh.harvard.eduAcademic Editor: Mauro Perretti

Publication date: 2011
Electronic publication date: 2011 Jan 5
Volume: 11
First page: 118
Last page: 120
Received 2010 Oct 5; Revised 2010 Nov 22; Accepted 2010 Dec 6
Copyright: Copyright © 2011 Suryasarathi Dasgupta.
Copyright year: 2011
License: This is an open access article distributed under the Creative Commons Attribution License, which permits unrestricted use, distribution, and reproduction in any medium, provided the original work is properly cited.
License URL: https://creativecommons.org/licenses/by/3.0/

Keywords: dendritic cells, inflammation, cytokine, toll-like receptor 2, polysaccharide A, immunoregulation

==============================
Directions in Science
*TheScientificWorldJOURNAL* (2011) 11, 118–120
ISSN 1537-744X; DOI 10.1100/tsw.2011.14

TheScientificWorldJOURNAL
www.thescientificworld.com

### Mediation of Immunoregulation by Dendritic Cells

Suryasarathi Dasgupta
*Channing Laboratory, Department of Medicine, Brigham and Women's Hospital, and Department of Microbiology and Molecular Genetics, Harvard Medical School, Boston*

**E-mail:** sdasgupta@rics.bwh.harvard.edu

*Received October 5, 2010; Revised November 22, 2010; Accepted December 6, 2010; Published January 5, 2011*

---

**KEYWORDS:** dendritic cells, inflammation, cytokine, toll-like receptor 2, polysaccharide A, immunoregulation

---

Inflammation is the body's natural response against insult from invasive infection or tissue injury. Persistence of inflammation usually leads to a pathological state. However, inflammation can be an important feature in physiology as well[1]. Immunological parameters that dictate or indicate the tissue's migration from a steady state to an inflamed state and back (restitution) need to be delineated extensively in order to enhance our overall understanding of homeostasis, and to design novel diagnostics and therapies to counter pathological disorders.

Induction of inflammation involves the participation of proinflammatory cells. Dendritic cells (DCs), with their capacity to produce high amounts of proinflammatory cytokines and inducing proinflammatory cytokine-producing T cells, would presumably play an important role in the process. A contrasting feature of DCs, that of immunoregulation or tolerance induction, has recently been elaborated, thereby pointing to a more complicated role of these cells in inflammatory response than initially thought. How DCs adjust to and presumably control the flux between the steady state and inflammatory state in a tissue might ultimately decide the fate of homeostasis of the corresponding tissue.

DCs are known to initiate an immune response by priming naïve T cells[2] and engendering an adaptive immune response. Properties in DCs such as antigen uptake, processing, presentation, maturation after sensing "danger signals", generation of proinflammatory cytokines (such as interleukin IL-12 and IL-23), up-regulation of surface molecules (such as costimulatory molecules [CD80, CD86, and CD40]), along with their ability to migrate to lymphoid organs and come in contact with T cells, ensures generation and sustenance of antigen-specific immune response. Unwanted immune response as observed in the case of autoimmune reaction leading to pathology can be speculated to involve several of these properties of DCs[3].

Induction of tolerance by DCs is generally considered as a function of antigen capture in the steady state[4]. Such DCs are often represented by immature DCs generated *in vitro* from circulating monocytes or bone-marrow precursors prior to stimulation with proinflammatory stimuli such as toll-like receptor (TLR) ligands, TNF-α, anti-CD40 mAb, or CD40 ligand. In addition to these steady-state properties of immature DCs, various DC programmings are being elaborated recently, which lead to tolerogenic responses by T cells[5]. The anti-inflammatory cytokine IL-10 produced by DCs has been suggested to induce a special set of regulatory T cells, called Tr1, which effect immunoregulation by further secreting

©2011 with author.

IL-10[6,7]. Interestingly, DCs from different tissues have been shown to have differential capacities in producing IL-10 and inducing immunoregulatory properties to T cells. DCs from Peyer's Patches (PP) in the mammalian intestine produced higher amounts of IL-10 in response to anti-CD40 stimulation than splenic DCs and, consequently, stimulated higher amounts of IL-10 and lower amounts of proinflammatory cytokine IFN-γ from naïve T cells[8,9]. Conversion of vitamin A to retinoic acid, utilizing enzymes called retinal dehydrogenase, is another strategy used by DCs to generate immunoregulatory T cells (Tregs) marked by FOX P3[10,11]. The generated retinoic acid then signals through the retinoic acid receptor on T cells and participates in the induction of FOX P3, a process mediated by the cytokine TGF-β in its activated form. Activation of TGF-β has been shown to be governed by a surface integrin on DCs, the $\alpha_v\beta_8$. Both in *in vitro* experiments and in mice deficient of this integrin on DCs, there was deficiency in the FoxP3+ T cells leading to autoimmunity and inflammation in the mice[12]. Indoleamine 2,3-dioxygease (IDO)[13,14] and the recently described β-catenin[15] further provide alternate pathways to induce Tregs by DCs.

Functional commitment of T-cell subsets dictating the fate of inflammation is a well-studied field in immunological research. Recently, DCs are also being classified based on what end of the spectrum they lie on in terms of inflammation. In the steady state, a specific subset of DCs found in gut and gut-associated lymphoid tissue (GALT) marked with surface molecule CD103 has been demonstrated to induce the extrathymic development of FOX P3+ Tregs[16,17]. However, DCs that do not express CD103 at the surface are not bereft of immunoregulatory capacities. Interestingly, a recent report from Laffont et al. showed that during intestinal inflammation, CD103+ DCs in MLN change their immunoregulatory character and add to the pathogenic outcome. Thus, CD103+ DCs in inflammation induce T cells to express lower FOX P3, but higher IFN-γ than their counterparts in the steady state[18]. Another class of DCs that have been shown to play an important role in tolerance are the plasmacytoid DCs (pDCs). pDCs are nonconventional DCs that have been previously reported to be the major cell type in producing interferon (IFNα/β) against viral attack. However, the literature demonstrating their role in immunoregulation is growing. pDCs have been shown to affect tolerance by inducing Tregs or T-cell anergy[19].

Another paradox in understanding how inflammatory responses are quenched during protection or resolution is the role played by surface receptors in the process. Pattern recognition receptors, which enable professional antigen-presenting cells like DCs to sense danger-associated patterns in order to invoke innate and adaptive immunity, might play an alternative role to precipitate immunoregulation. Recently, one such receptor, TLR2, has been implicated in controlling autoimmune disorders in various tissues. Manicassamy et al. showed that TLR2 ligation by fungal-derived molecules on DCs results in an anti-inflammatory effect, resulting in control of a mouse model of multiple sclerosis or EAE[20]**.** In separate studies, it was demonstrated that TLR2 deficiency fails to protect mice from EAE or from colitis by a immune-protective polysaccharide, polysaccharide A (PSA), from the capsule of commensal flora *Bacteroides fragilis*[21,22]. PSA has previously been shown to be a ligand for TLR2 on antigen-presenting cells and invoke usual proinflammatory cytokine IFN-γ from T cells[23]. Thus, it becomes important to delineate mechanisms that involve similar elements and compartments of the immune system to invoke contrasting outcomes.

### REFERENCES

1. Medzhitov, R. (2008) Origin and physiological roles of inflammation. *Nature* **454(7203),** 428–435.
2. Banchereau, J. and Steinman, R.M. (1998) Dendritic cells and the control of immunity. *Nature* **392(6673),** 245–252.
3. Steinman, R.M. and Banchereau, J. (2007) Taking dendritic cells into medicine. *Nature* **449(7161),** 419–426.
4. Steinman, R.M., Hawiger, D., and Nussenzweig, M.C. (2003) Tolerogenic dendritic cells. *Annu. Rev. Immunol.* **21,** 685–711.
5. Pulendran, B., Tang, H., and Manicassamy, S. (2010) Programming dendritic cells to induce T(H)2 and tolerogenic responses. *Nat. Immunol.* **11(8),** 647–655.

6. Gregori, S. et al. (2010) Differentiation of type 1 T regulatory cells (Tr1) by tolerogenic DC-10 requires the IL-10-dependent ILT4/HLA-G pathway. *Blood* **116(6),** 935–944.
7. Battaglia, M. et al. (2006) Tr1 cells: from discovery to their clinical application. *Semin. Immunol.* **18(2),** 120–127.
8. Iwasaki, A. and Kelsall, B.L. (1999) Freshly isolated Peyer's patch, but not spleen, dendritic cells produce interleukin 10 and induce the differentiation of T helper type 2 cells. *J. Exp. Med.* **190(2),** 229–239.
9. Iwasaki, A. and Kelsall, B.L. (2001) Unique functions of CD11b+, CD8 alpha+, and double-negative Peyer's patch dendritic cells. *J. Immunol.* **166(8),** 4884–4890.
10. Mora, J.R., Iwata, M., and von Andrian, U.H. (2008) Vitamin effects on the immune system: vitamins A and D take centre stage. *Nat. Rev. Immunol.* **8(9),** 685–698.
11. Sun, C.M. et al. (2007) Small intestine lamina propria dendritic cells promote de novo generation of Foxp3 T reg cells via retinoic acid. *J. Exp. Med.* **204(8),** 1775–1785.
12. Travis, M.A. et al. (2007) Loss of integrin alpha(v)beta8 on dendritic cells causes autoimmunity and colitis in mice. *Nature* **449(7160),** 361–365.
13. Manches, O. et al. (2008) HIV-activated human plasmacytoid DCs induce Tregs through an indoleamine 2,3-dioxygenase-dependent mechanism. *J. Clin. Invest.* **118(10),** 3431–3439.
14. Chen, W. et al. (2008) The indoleamine 2,3-dioxygenase pathway is essential for human plasmacytoid dendritic cell-induced adaptive T regulatory cell generation. *J. Immunol.* **181(8),** 5396–5404.
15. Manicassamy, S. et al. (2010) Activation of beta-catenin in dendritic cells regulates immunity versus tolerance in the intestine. *Science* **329(5993),** 849–853.
16. Coombes, J.L. et al. (2007) A functionally specialized population of mucosal CD103+ DCs induces Foxp3+ regulatory T cells via a TGF-beta and retinoic acid-dependent mechanism. *J. Exp. Med.* **204(8),** 1757–1764.
17. Coombes, J.L. and Powrie, F. (2008) Dendritic cells in intestinal immune regulation. *Nat. Rev. Immunol.* **8(6),** 435–446.
18. Laffont, S., Siddiqui, K.R., and Powrie, F. (2010) Intestinal inflammation abrogates the tolerogenic properties of MLN CD103+ dendritic cells. *Eur. J. Immunol.* **40(7),** 1877–1883.
19. Swiecki, M. and Colonna, M. (2010) Unraveling the functions of plasmacytoid dendritic cells during viral infections, autoimmunity, and tolerance. *Immunol. Rev.* **234(1),** 142–162.
20. Manicassamy, S. et al. (2009) Toll-like receptor 2-dependent induction of vitamin A-metabolizing enzymes in dendritic cells promotes T regulatory responses and inhibits autoimmunity. *Nat. Med.* **15(4),** 401–409.
21. Ochoa-Reparaz, J. et al. (2010) A polysaccharide from the human commensal Bacteroides fragilis protects against CNS demyelinating disease. *Mucosal Immunol.* **3(5),** 487–495.
22. Round, J.L. and Mazmanian, S.K. (2010) Inducible Foxp3+ regulatory T-cell development by a commensal bacterium of the intestinal microbiota. *Proc. Natl. Acad. Sci. U. S. A.* **107(27),** 12204–12209.
23. Wang, Q. et al. (2006) A bacterial carbohydrate links innate and adaptive responses through Toll-like receptor 2. *J. Exp. Med.* **203,** 2853–2863.

**This article should be cited as follows**:

Dasgupta, S. (2011) Mediation of immunoregulation by dendritic cells. *TheScientificWorldJOURNAL* **11,** 118–120. DOI 10.1100/tsw.2011.14.
